# Direct coronary stenting in reducing radiation and radiocontrast consumption

**DOI:** 10.2478/v10019-010-0016-x

**Published:** 2010-09-09

**Authors:** Jasmin Caluk, Enes Osmanovic, Fahir Barakovic, Zumreta Kusljugic, Ibrahim Terzic, Selma Caluk, Amela Sofic

**Affiliations:** 1 BH Heart Center Tuzla, Department of Interventional Cardiology, Tuzla, Bosnia-Herzegovina; 2 University Clinical Center Tuzla, Department of Internal Medicine, Tuzla, Bosnia-Herzegovina; 3 Clinical Center of Sarajevo University, Department of Radiology, Sarajevo, Bosnia-Herzegovina

**Keywords:** coronary stenting, balloon predilatation, percutaneous transluminal coronary angioplasty, expenses

## Abstract

**Introduction:**

Coronary stenting is the primary means of coronary revascularization. There are two basic techniques of stent implantation: stenting with balloon predilatation of stenosis and stenting without predilatation (direct stenting). Limiting the time that a fluoroscope is activated and by appropriately managing the intensity of the applied radiation, the operator limits radiation in the environment, and this saves the exposure to the patient and all personnel in the room. Nephrotoxicity is one of the most important properties of radiocontrast. The smaller amount of radiocontrast used also provides multiple positive effects, primarily regarding the periprocedural risk for the patients with the reduced renal function. The goal of the study was to compare fluoroscopy time, the amount of radiocontrast, and expenses of material used in direct stenting and in stenting with predilatation.

**Patients and methods:**

In a prospective study, 70 patients with coronary disease were randomized to direct stenting, or stenting with predilatation.

**Results:**

Fluoroscopy time and radiocontrast use were significantly reduced in the directly stented patients in comparison to the patients stented with balloon-predilatation. The study showed a significant reduction of expenses when using a direct stenting method in comparison to stenting with predilatation.

**Conslusions:**

If the operator predicts that the procedure can be performed using direct stenting, he is encouraged to do so. Direct stenting is recommended for all percutaneous coronary interventions when appropriate conditions have been met. If direct stenting has been unsuccessful, the procedure can be converted to predilatation.

## Introduction

Heart is supplied with blood through coronary arteries. Disbalance in myocardial oxygen supply and demand may cause myocardial ischemia with contractile dysfunction, arrhythmias, infarction, and possibly death.^1^ Interventional cardiology deals with catheter-based interventions in the treatment of the structural heart disease. Coronary stent implantation is the primary means of coronary revascularization.^2^ Stenting of arterial stenosis in other organs is also the method of choice as the minimally invasive interventional procedure.^3^ There are two basic techniques of the coronary stent implantation. The first one consists of PTCA (percutaneous transluminal coronary angioplasty)-balloon predilatation of stenosis before stenting, a sort of ‘preparing the ground’ for stenting itself. This technique is the usual, or the conventional one, evolved from bail-out stenting used to treat complications, such as coronary dissection, in the era of PTCA. The second technique is somewhat newer. It implies stent implantation without pre-dilatation, and is called ‘direct stenting’.

Fluoroscopic radiation is a carcinogen that can also cause a severe injury (radiation burns) to patients and practitioners.^4^ When fluoroscopy is well managed, the likelihood that these severe effects could occur is extremely low. Medical practitioners who have accumulated considerable radiation doses have been shown to have developed radiation-induced cancers, cataracts, or skin injury.^5^–^7^ Attention to rigorous radiation abatement measures is therefore warranted and required.^8^ Time, intensity, distance, and shielding (TIDS) describes the management of the radiation exposure by minimizing the time to which one is exposed to radiation, by minimizing the intensity of the radiation that is deployed, by maximizing the distance from the source, and by shielding the personnel from the radiation. The operator can limit the radiation in the cathlab by limiting the time that the fluoroscope is activated and by appropriately managing the intensity of the applied radiation, thus reducing the exposure to the patient and all personnel in the room. Cardioangiographic equipment is one of the most sophisticated and complex equipment used in medicine.^4^ The goal in cardioangiographic imaging is to produce an X-ray beam that results in an excellent compromise between the appropriate image quality and the radiation dose. Limiting the beam-on time limits the exposure time for the patient, but also for the personnel.

Blood vessel walls and myocardium have similar X-ray absorbance to that of blood, making their imaging by conventional radiographic techniques virtually impossible without the use of the intravascular contrast agent. Therefore, the use of radiocontrast is absolutely necessary in order to obtain images of coronary arteries. Those images are obtained by directly injecting the radiocontrast into the coronaries and recording an X-ray image, or sequence. Adverse reactions to radiocontrast are most importantly anaphylactoid, but also toxic effects, such as nausea or vomiting, but the incidence of adverse reactions has been significantly reduced with the use of nonionic contrast media.^9^ Nephrotoxicity is one of the most important properties of radiocontrast. The smaller amount of radiocontrast used also provides multiple positive effects, primarily regarding the periprocedural risk for the patients with the reduced renal function. Contrast induced nephropathy (CIN) causes renal failure, increased morbidity, prolonged hospital stay, higher hospitalization costs, and increased mortality.^10^ Although its pathogenesis remains unclear, CIN is probably due to a combination of decreased renal medullary perfusion (possibly because of alterations in renin-angiotensin system, nitric oxide synthesis, adenosine metabolism, prostacyclin production, and endothelin synthesis)^11^–^13^, resulting in critical medullary ischemia and direct tubular toxicity.^14^ Patients with diabetic nephropathy are at especially great risk from CIN. Although it is logical to assume that the risk is reduced when using smaller amounts of contrast per procedure, there is no consensus regarding a ‘safety dose’. Additional benefits from the reduced radiocontrast use are primarily economical, for these agents are relatively expensive.

The goals of the study were:

To compare fluoroscopy time as a measure of radiation exposure during direct coronary stenting and stenting with predilatation.
To compare the amount of radiocontrast used during direct coronary stenting and stenting with predilatation.
To compare expenses of material used in direct stenting and in stenting with predilatation.

## Patients and methods

In a prospective study, 70 patients that have undergone coronary stent implantation as the treatment of coronary disease were analyzed. The patients were randomized into two groups of 35 patients each. The patients in one group were treated by stenting with PTCA-balloon predilatation, and the patients in the other group – by direct stenting. Groups were similar by the criteria of age, gender, affected coronary arteries, types of stents used for the treatment, and severity of coronary stenoses. Exclusion criteria were: acute myocardial infarction, two or more stenoses treated per patient per procedure, and chronic total coronary occlusions on coronary angiography finding.

All patients have undergone prior selective coronary angiography. Thereafter, the patients randomized to conventional stenting had their coronary stents implanted after PTCA-balloon predilatation, and those randomized to direct stenting had their stents implanted directly into coronary lesions, without the prior PTCA-balloon predilatation.

Fluoroscopy time measured in seconds and radiocontrast dye use measured in milliliters were recorded for all patients. Selective coronary angiography and percutaneous coronary interventions (PCIs) were performed on cardioangiograph Siemens Axiom.

## Results

There were no significant differences in age (p=0.17) or gender (p=0.51) between the groups. The average age in directly stented group was 57.40±10.03, and that in the conventionally stented group 54.31±8.70. The most often affected coronary artery was left anterior descending coronary artery in both groups (p=0.23). There was no significant difference in stenosis intensity between the groups (Table 1).

During the study, no ECG showed signs of newly onset myocardial necrosis (new Q-wave), nor new bundle-branch blocks, which would speak in favor of significant myocardial necrosis.

Fluoroscopy time and radiocontrast use were significantly reduced in patients that were stented directly, in comparison to patients stented with prior balloon-predilatation (Table 2).

In both groups, using quantitative coronary angiography (QCA), we found a complete elimination of previous stenosis to 0% in all patients, without residual stenoses. We found no major adverse cardiac events (MACE), defined as urgent coronary revascularization, myocardial infarction, lethal outcome in either of the groups.

A financial analysis of the expenses of materials used during direct stenting, and stenting with pre-dilatation showed an average reduction of costs of 27.86±2.81 % (p<0.05) when using the direct stenting method in comparison to stenting with predilatation. The basic role in this cost reduction plays the elimination of the use of PTCA balloon for predilatation and lower amount of radiocontrast used in direct stenting.

## Discussion

During 1993 two important trials compared the implantation of Palmaz-Schatz coronary stents to conventional PTCA, and established coronary stenting as the standard treatment. The BENESTENT trial involved 520, and the STRESS trial 410 patients, independently demonstrating that coronary stents reduce restenoses (>50% of new stenosis of the earlier treated artery at the site of treatment – PTCA or stenting).^15^,^16^ As early as 1999, stenting took 84.2% of all PCIs.^17^ Direct stenting is defined as positioning and implantation of stent without balloon-predilatation of coronary stenosis.^18^ It is a new strategy of the coronary disease treatment enabled by the development of the advanced stent and the implantation system design with the low cross-section area, high safety standards, and high rated burst pressures.^19^ Initial registers show a high success rate in combination with low complication rates.^20^ The procedure is safe in selected cases, and can help reducing the expense of coronary interventions through the reduction in total procedural and fluoroscopy time, the amount of radiocontrast and the number of angiographic catheters used.^21^,^22^ Wilson *et al.* showed that direct stenting has positive effects on total procedural time, radiation exposure, and the use of radiocontrast.^23^ In our research, direct stenting has significant positive effects regarding these criteria too, in comparison to stenting with predilatation. The procedural outcome seems to be superior without predilatation, because of the reduced incidence of coronary dissections at stent edges.^24^ In our study, the procedural outcome was the same in both groups: the reduction of coronary stenoses, measured by quantitative coronary angiography, was complete to 0% in all patients, without residual stenoses. We have found no edge-dissections as a periprocedural complication. In the group of patients treated by stenting with predilatation, 2 out of 35 patients had localized small, non-obstructive coronary dissections after balloon-predilatation, which were routinely covered by stent implantation immediately afterwards. In the directly stented group, out of 35 patients, there were no coronary dissections after stenting. In the DISCO trial, conducted in 10 centers in Spain, 416 patients with 446 coronary lesions were randomized to conventional or direct stenting.^25^ The main goal of this trial was to evaluate safety, feasibility, and the effect on angiographical restenosis of direct stenting in comparison to the conventional method of stenting with predilatation. The direct stenting strategy was effectively accomplished in 97% of lesions, and the patients converted to the predilatation strategy were all treated successfully. In our research (on a much smaller sample), the direct stenting strategy was successfully accomplished in all patients of that group, and there was no conversion to the pre-dilatation strategy. Of course, it is only realistic to assume that a randomized sample that would include a larger number of patients would result in a certain small number of patients to be converted to predilatation, which we observe sometimes in our daily practice, especially in patients with critical sub-occlusions or highly calcified lesions. In our study, we found no MACE in either group. We have also found no major peri- or postprocedural complications. There were no electrocardiographical signs of myocardial necrosis, periprocedurally, or during the one-month follow-up.

The reduction of fluoroscopy time is very important both for the patient and for the staff. Ionizing radiation is one of the leading causes of malignancies^26^, and the staff, especially the operator who is close to the radiation source and the source of scattered radiation during the entire intervention, is under a significant health risk.^27^ According to our study, the fluoroscopy time was significantly reduced during direct stenting (p=0.0001) in comparison to conventional stenting, regardless of the artery treated. While we found fluoroscopy time to be 392.8±207.7 s during stenting with predilatation, we observed that this value in direct stenting was 204.1±98.46 s. In the study of Martinez-Elbal *et al.*, the most important differences between the patients treated by direct or conventional stenting were significant fluoroscopy time reduction in direct stenting when compared to stenting with pre-dilatation (6.4 min: 9.2min, respectively, p<0.0005) and significant reduction of total procedural time (21.2 min: 27.8 min, respectively, p<0.0005).^25^ The interventional radiation environment creates the conditions for accumulation of high doses in the staff. That is why it is essential to pay attention to rigorous measures of decreasing radiation exposure.^8^

The smaller amount of radiocontrast used also provides multiple positive effects, primarily regarding the periprocedural risk for the patients with reduced renal function. The main adverse effects from radiocontrast use are anaphylactic reactions and contrast-induced nephropathy (CIN). The use of radiocontrast, measured in milliliters, in our research was significantly lower in direct stenting, than in stenting with predilatation (280 (100–350) : 350 (200–400), respectively, p=0.0001). We found no CIN in either of the groups. Additional benefits from the reduced radiocontrast use are primarily economical, for these agents are relatively expensive.

With the elimination of expenses by saving PTCA-balloons and their dilatation catheters, the reduction in radiocontrast use also significantly reduces the overall cost of the intervention. In our study, a 27.86±4.81 % (p<0.05) cost reduction was recorded when using direct stenting in comparison to stenting with predilatation. The expenses related to the material used were taken into account. This sort of cost saving is similar to the reports of other authors, who describe cost savings in the range 20%–40% with the use of direct stenting.^18^

## Conclusions

Direct stenting is defined as positioning and implantation of coronary stents without prior balloon dilatation of coronary stenosis. It can be used to accelerate the procedure and reduce intimal trauma. Complex lesions in small arteries and severe calcifications limit the use of this technique. Direct stenting reduces health risks connected to radiation by significantly reducing fluoroscopy time. It also decreases the risk of contrast induced nephropathy by using significantly less radiocontrast when compared to stenting with predilatation. Direct stenting is not connected to an increased risk of major adverse cardiac events (acute myocardial infarction, urgent coronary revascularization, lethal outcome) in comparison to stenting with pre-dilatation. New low-profile stents with high rated burst pressure values have enabled the routine use of this PCI technique. If direct stenting has been unsuccessful, the procedure can be converted to predilatation. If the operator predicts that the procedure can be performed using direct stenting, he is encouraged to do so. Direct stenting is recommended for all percutaneous coronary interventions when appropriate conditions have been met.

## Figures and Tables

**TABLE 1:** Intensity of coronary stenoses, observed by coronary arteries in investigated groups

**Characteristics**		**Stenting method**				
		**DS n = 35**		**SWP n = 35**		**p**
**Artery**		n	% stenosis	n	% stenosis	
	
	-RCA	9	86.11 ± 6.50	7	86.42 ± 5.56	0.91
	-LAD	21	84.28 ± 6.18	17	87.64 ± 5.62	0.09
	-CX	5	83.00 ± 6.70	11	87.72 ± 5.64	0.16

Legend: RCA – right coronary artery, LAD – left anterior descending coronary artery, CX – circumflex coronary artery, DS – direct stenting; SWP – stenting with predilatation. Values are displayed as mean, standard deviation, and in absolute numbers.

**TABLE 2:** Amount of radiocontrast used and fluoroscopy time in investigated groups

Paremeters:		Stenting method		
		DS n = 35	SWP n = 35	p
Fluoroscopy time	s	204.1 ± 98.46	392.8 ± 207.7	^‡*^0.0001
Radiocontrast	ml	280 (100 – 350)	350 (200 – 400)	^⋄^0.0001

Legend: s-second, ml-millilitre; DS – direct stenting; SWP – stenting with predilatation;

‡* Student T-test (df 68, test statistic 4.85, p < 0.0001);

⋄ Mann-Whitney test (Large sample test statistic Z −4.58; p < 0.0001).
